# Commercial Versus Medicaid Insurance and Use of High-Priced Anticancer Treatments

**DOI:** 10.1093/oncolo/oyae035

**Published:** 2024-03-14

**Authors:** Aaron P Mitchell, Alan C Kinlaw, Sharon Peacock-Hinton, Stacie B Dusetzina, Aaron N Winn, Hanna K Sanoff, Jennifer L Lund

**Affiliations:** Department of Epidemiology and Biostatistics, Memorial Sloan Kettering Cancer Center, New York, NY, United States; Department of Medicine, Memorial Sloan Kettering Cancer Center, New York, NY, United States; Cecil G. Sheps Center for Health Services Research, University of North Carolina at Chapel Hill, Chapel Hill, NC, United States; Cecil G. Sheps Center for Health Services Research, University of North Carolina at Chapel Hill, Chapel Hill, NC, United States; Division of Pharmaceutical Outcomes and Policy, Eshelman School of Pharmacy, University of North Carolina at Chapel Hill, Chapel Hill, NC, United States; Department of Epidemiology, Gillings School of Global Public Health, University of North Carolina at Chapel Hill, Chapel Hill, NC, United States; Department of Health Policy, Vanderbilt University School of Medicine, Nashville, TN, United States; Vanderbilt-Ingram Cancer Center, Nashville, TN, United States; University of Illinois at Chicago, Chicago, IL, United States; Department of Hematology/ Oncology, School of Medicine, University of North Carolina at Chapel Hill, Chapel Hill, NC, United States; Lineberger Comprehensive Cancer Center, University of North Carolina at Chapel Hill, Chapel Hill, NC, United States; Department of Epidemiology, Gillings School of Global Public Health, University of North Carolina at Chapel Hill, Chapel Hill, NC, United States; Lineberger Comprehensive Cancer Center, University of North Carolina at Chapel Hill, Chapel Hill, NC, United States

**Keywords:** antineoplastic agents, practice pattern, clinical, reimbursement, incentive, fee-for-service plans, insurance coverage

## Abstract

**Background:**

Because the markups on cancer drugs vary by payor, providers’ financial incentive to use high-price drugs is differential according to each patient’s insurance type. We evaluated the association between patient insurer (commercial vs Medicaid) and the use of high-priced cancer treatments.

**Materials and Methods:**

We linked cancer registry, administrative claims, and demographic data for individuals diagnosed with cancer in North Carolina from 2004 to 2011, with either commercial or Medicaid insurance. We selected cancers with multiple FDA-approved, guideline-recommended chemotherapy options and large price differences between treatment options: advanced colorectal, lung, and head and neck cancer. The outcome was a receipt of a higher-priced option, and the exposure was insurer: commercial versus Medicaid. We estimated risk ratios (RRs) for the association between insurer and higher-priced treatment using log-binomial models with inverse probability of exposure weights.

**Results:**

Of 812 patients, 209 (26%) had Medicaid. The unadjusted risk of receiving higher-priced treatment was 36% (215/603) for commercially insured and 27% (57/209) for Medicaid insured (RR: 1.31, 95% CI: 1.02-1.67). After adjustment for confounders the association was attenuated (RR: 1.15, 95% CI: 0.81-1.65). Exploratory subgroup analysis suggested that commercial insurance was associated with increased receipt of higher-priced treatment among patients treated by non-NCI-designated providers (RR: 1.53, 95% CI: 1.14-2.04).

**Conclusions:**

Individuals with Medicaid and commercial insurance received high-priced treatments in similar proportion, after accounting for differences in case mix. However, modification by provider characteristics suggests that insurance type may influence treatment selection for some patient groups. Further work is needed to determine the relationship between insurance status and newer, high-price drugs such as immune-oncology agents.

## Teaser

This study evaluated the association between insurer (commercial vs Medicaid) and the use of high-priced cancer treatments. Commercially insured and Medicaid-insured patients received high-priced treatments in similar proportion overall, but commercially insured patients were more likely to receive high-priced treatments in community practice settings.

**Implications for Practice**

Commercial insurers reimburse for cancer drugs at higher rates than public insurers such as Medicaid, and whether this results in differential access to high-priced cancer treatments is unknown. Results of this study showed that commercially insured and Medicaid-insured patients received high-priced treatments in similar proportion, after accounting for patient factors. However, in community settings, where physician compensation is more often tied to billing than in academic settings, commercially insured patients were more likely to receive high-priced treatments, suggesting potential responsiveness to financial incentives.

## Background

The current payment model for most infused anticancer therapies, termed the “buy and bill” model, creates an adverse incentive for providers to prefer higher-price drugs.^1^ Under this model, provider reimbursement increases with drug prices. Public payers, including Medicare and many Medicaid programs including NC Medicaid, pay for clinician-administered drugs at the average sales price plus an add-on payment that is a percentage of the drug’s price (currently 4.3% until 2027 for most public payers due to sequestration cuts).^2-4^ Higher-priced drugs return higher absolute dollar amounts to a practice under this formula.

Commercial insurers also reimburse in proportion to drug price and generally pay much more than public payers.^5-7^ The median add-on payment negotiated with commercial payers at major hospitals was found to range from +118% to as high as +634% above average sales price for some cancer drugs.^7^ For example, for a 700 mg bevacizumab infusion, assuming a hospital acquired the drug at ASP (approximately $4623), the hospital would anticipate retaining a $277 markup at Medicare rates and a $7490 markup at the median negotiated commercial rate.^7^ Accordingly, providers have a greater financial incentive to use higher-priced drugs for their commercially insured patients relative to their publicly insured patients.^8^

To date, there has been little work to establish whether, and to what degree, physicians respond to this incentive in real-world practice. A systematic review found consistent evidence that providers do respond to financial incentives in terms of what care they deliver to people with cancer, including which anticancer drugs are chosen.^9^ Prior studies have also suggested that oncologists may be responsive to financial incentives, observing a greater use of high-margin but low-value cancer treatments in care settings wherein physician compensation is more likely to be directly tied to billing rather than salaried; physician office (vs hospital outpatient)^10-12^ and nonacademic (vs academic) settings.^13^ However, whether there are differences in the use of high-priced cancer drugs on the basis of patient insurance has not been studied. The greater financial incentive among commercially insured patients would suggest that providers may be more likely to recommend high-price drugs to commercially insured patients. An additional consideration regarding cancer care delivery with respect to patient insurance is the worse outcomes observed among Medicaid-insured patients with cancer,^14,15^ raising questions about whether Medicaid-insured patients enjoy fully equal access to high-quality cancer treatment, which may include high-priced drugs. The goal of this study was to determine whether commercially insured versus Medicaid-insured patients with cancer are similarly likely to receive treatment with expensive anticancer drugs.

## Methods

### Patient Population and Physician Assignment

The primary data source was the North Carolina Central Cancer Registry, linked to state Medicaid and commercial insurance claims from 2004 to 2011. We included patients aged 18-64 years, newly diagnosed with a cancer of interest, received a treatment regimen of interest within 120 days of diagnosis (see below), and had continuous enrollment in either commercial insurance or Medicaid for 180 days prior to diagnosis through 60 days after their first treatment claim. We excluded patients who were Medicare-eligible, had a prior cancer diagnosis within 5 years, or had missing data for cancer type, insurance, or diagnosis date (see Table 1 showing patient selection).

**Table 1.** Patient selection by cancer type, total, and by cancer type.

	Number remaining (% of starting)			
	Total	Colorectal	Head and neck	Lung
Newly diagnosed stage IV colorectal (2004-2011), stages II-IV head and neck (2006-2011), or stage IV lung adenocarcinoma (2006-2011), age 18-64, with insurance coverage at time of diagnosis	3840 (100)	954 (100)	1345 (100)	1541 (100)
Received any component of a high- or low-cost treatment regimen within 120 days of diagnosis	1556 (40.5)	508 (53.2)	526 (39.1)	522 (33.9)
Received all components of a high- or low-cost treatment regimen within 60 days of starting treatment	1531 (39.9)	501 (52.5)	525 (39.0)	505 (32.8)
Continuous insurance enrollment for 180 days before and 60 days after starting treatment	812 (21.1)	263 (27.6)	311 (23.1)	238 (15.4)

### Cohort and Outcome Definitions

The cancer types of interest included those we identified as having several different treatment options that were FDA-approved and recommended by the National Comprehensive Cancer Network (NCCN) during the study period, and at least one of the treatment options included a high-price drug and at least one other treatment option that did not. Following prior work,^13^ we defined a set of common treatment options of interest that either did (termed “high-price”) or did not (termed “low-price”) contain an infused biologic agent. The cancer types and treatment categories selected were stage IV colorectal (low-price = FOLFOX or FOLFIRI, high-price = [FOLFOX or FOLFIRI] + [bevacizumab or cetuximab or panitumumab]), stages II-IV head and neck cancer (low-price = any cytotoxic agent, high-price = cetuximab ± any cytotoxic agent), stage IV lung adenocarcinoma (low-price = [cisplatin or carboplatin] + [paclitaxel, nab-paclitaxel, or pemetrexed], high-price = [cisplatin or carboplatin] + [paclitaxel, nab-paclitaxel, or pemetrexed] + bevacizumab). We included patients diagnosed 2004-2011 for colorectal and 2006-2011 for head and neck and lung, selected to reflect the time period immediately after the high-price drug was first approved and recommended alongside low-price treatment options for each cancer type (see Supplementary Table S1 for a detailed description of cancer selection and classification of treatment regimens).

The primary outcome was receipt of one of the defined high-price treatment options. The outcome was defined by drugs received during the first 60 days after beginning chemotherapy. This period was chosen to appropriately identify patients who received a biologic agent (eg, bevacizumab) as part of their first line of treatment but had delayed initiation of that agent due to recent surgery and minimize the inclusion of any drugs given as the second line of treatment.

### Exposure Definition

The primary exposure was patient primary insurance type, Medicaid versus commercial insurance, identified by the source of claims for each patient.

### Potential Confounders

Potential confounding variables included cancer type, location of initial cancer treatment (physician office vs hospital outpatient),^16,17^ treatment by a provider practicing in an NCI-designated Comprehensive Cancer Center (NCI vs non-NCI, defined by the ZIP code billing location of treatment claims, following prior work),^13^ patient sex, race (White/non-White), age at diagnosis, calendar year of diagnosis, county-level poverty (US Census data), and comorbidities. We included comorbidities that (1) present relative contraindications to the specific anticancer agents included, defined as health conditions listed as a “contraindication” or FDA boxed (“black box”) warning on the manufacturer label, or (2) are associated with patient frailty based on a validated claims-based algorithm^18,19^ (see Supplementary Table S2 for included conditions). Comorbidities were assessed during the 180-day period prior to first cancer treatment claim.

### Statistical Analysis

We used stabilized inverse probability of exposure (propensity score) weighting to minimize potential confounding by the aforementioned variables.^20,21^ We assessed covariate balance between exposure groups by calculating the standardized mean difference both before and after weighting.^22^

We used log-binomial regression models to estimate the prevalence ratio for receiving high-price treatment, comparing individuals covered by commercial versus Medicaid insurance. To account for confounding variables, we ran weighted models and estimated 95% CIs using a robust variance estimator.

In exploratory analyses, we assessed for potential interaction by conducting stratified analyses by 3 variables of interest: location of initial cancer treatment, provider NCI designation, and cancer type. Due to limited sample size within subgroups, we selected a smaller set of covariates for balancing using inverse probability of exposure weighting. These covariates were prioritized based on our theoretical model, as those most likely to result in substantial confounding. The resulting set of covariates selected for balancing within each of the stratified analyses were (1) location of initial treatment: cancer type, diagnosis year, age at diagnosis, recent surgery, gastrointestinal bleeding or hemoptysis, and brain metastasis; (2) provider NCI designation: cancer type and diagnosis year; and (3) cancer type: diagnosis year, age at diagnosis, recent surgery, and gastrointestinal bleeding or hemoptysis (see Supplementary Table S3 for covariates included within each subgroup analysis).

Data management and statistical analyses were conducted using SAS version 9.4 (SAS Institute, Inc., Cary, NC).

## Results

Our cohort included 812 patients with colorectal, head and neck, or lung adenocarcinoma (Table 1). 74.3% (*n* = 603) had commercial insurance and 25.7% (*n* = 209) had Medicaid insurance (Table 2). Regarding cancer type, 38.3% (*n* = 311) had head and neck cancer, 32.4% (*n* = 263) had colorectal cancer, and 29.3% (*n* = 238) had lung cancer. Most (63.3% [*n* = 514]) received treatment in the physician’s office setting; 36.7% (*n* = 298) received treatment in the hospital outpatient setting.

**Table 2.** Cohort characteristics, overall and by insurance type.

	All patients (*N* = 812)	Medicaid (*n* = 209, 25.7%)	Commercial (*n* = 603, 74.3%)
Female gender, *n* (%)	310 (38.2)	81 (38.8)	229 (38.0)
Median age in years (IQR)	54 (49-59)	54 (47-59)	55 (50-59)
Non-White race, *n* (%)	184 (22.7)	107 (51.2)	77 (12.8)
Median poverty prevalence in patient’s county (IQR)	14 (12-17)	16 (13-21)	14 (11-16)
Year of diagnosis, *n* (%)			
2004-2005	52 (6.4)	12 (5.7)	40 (6.6)
2006-2007	189 (23.3)	43 (20.6)	146 (24.2)
2008-2009	302 (37.2)	107 (51.2)	195 (32.3)
2010-2011	269 (33.1)	47 (22.5)	222 (36.8)
Provider NCI designation, *n* (%)			
NCI	163 (20.1)	27 (12.9)	136 (22.6)
Non-NCI	637 (78.4)	181 (86.6)	456 (75.6)
Unknown	12	<11	<11
Cancer type, *n* (%)			
Colorectal	263 (32.4)	54 (25.8)	209 (34.7)
Head and neck	311 (38.3)	106 (50.7)	205 (34)
Lung	238 (29.3)	49 (23.4)	189 (31.3)
Location of first treatment day, *n* (%)			
Hospital outpatient	298 (36.7)	79 (37.8)	219 (36.3)
Physician office	514 (63.3)	130 (62.2)	384 (63.7)
Number of drug contraindications, *n* (%)			
0	290 (35.7)	73 (34.9)	217 (36.0)
1	356 (43.8)	74 (35.4)	282 (46.8)
≥2	166 (20.4)	62 (29.7)	104 (17.2)
Selected contraindications, *n* (%)			
Recent surgery	355 (43.7)	96 (45.9)	259 (43.0)
Gastrointestinal bleeding or hemoptysis	160 (19.7)	54 (25.8)	106 (17.6)
Brain metastasis	76 (9.4)	16 (7.7)	60 (10.0)
Number of frailty indicators, *n* (%)			
0	541 (66.6)	109 (52.2)	432 (71.6)
1	150 (18.5)	45 (21.5)	105 (17.4)
≥2	121 (14.9)	55 (26.3)	66 (10.9)

Drug contraindications and frailty indicators are shown in tabulated form because of low frequencies of many individual conditions which may result in possible reidentification due to cell sizes <11; selected contraindications with the greatest theorized potential to affect physician selection of high-price versus low-price treatments are shown individually.

Prior to propensity score weighting, several covariates were unbalanced comparing the Medicaid-insured versus commercially insured cohorts (see Supplementary Table 4 for analysis of covariate balance). Several notable examples include the proportion of each cohort with non-White race (51.2% of Medicaid-insured vs 12.8% of commercially insured, SMD = 0.9), colorectal cancer (25.8% vs 34.7%, SMD = 0.19), head and neck cancer (50.7% vs 34.0%, SMD = 0.34), lung cancer (23.4% vs 31.3%, SMD = 0.18), number of drug contraindications ≥2 (29.7% vs 17.2%, SMD = 0.30), and number of frailty indicators ≥2 (26.3% vs 10.9%, SMD = 0.4). Covariate balance was improved after propensity score weighting, with the only covariates having a residual SMD > 0.1 being proportion living in counties with poverty prevalence <12% (19.5% vs 26.3%, SMD = 0.16) and proportion living in counties with poverty prevalence ≥12% but <14% (36.1% vs 22.7%, SMD = 0.30).

Among patients received treatment, a greater proportion of commercially insured than Medicaid-insured patients received high-price treatment (35.7% vs 27.5%; Table 3). The magnitude of this difference was attenuated after propensity score weighting (35.3% vs 30.5%).

**Table 3.** Unadjusted and weighted prevalence of high versus low-price treatment, by insurance type.

	Unadjusted			Weighted	
	*n*	HP treatment	LP treatment	HP treatment	LP treatment
Overall, *n* (%)	812	272 (33.5)	540 (66.5)	276.0 (33.9)	537.1 (66.1)
Insurance type, *n* (%)					
Commercial	603	215 (35.7)	388 (64.3)	206.5 (35.3)	379.0 (64.7)
Medicaid	209	57 (27.3)	152 (72.7)	69.5 (30.5)	158.1 (69.5)

Abbreviations: HP, high price; LP, low cost.

Commercial insurance was associated with greater likelihood of receiving high-price treatment in the unweighted cohort (relative risk vs Medicaid insurance: 1.31, 95% CI: 1.02-1.67) but not in the weighted cohort (1.15, 95% CI: 0.81-1.65; Table 4).

**Table 4.** Relative risk of high-price treatment for commercially insured versus Medicaid-insured patients, in unadjusted and weighted data, overall cohort and within exploratory subgroups.

	Unadjusted		Weighted
	n	RR (95% CI)	RR (95% CI)
Overall	812	1.31 (1.02-1.67)	1.15 (0.81-1.65)
Cancer type			
Colorectal	263	1.42 (1.02-1.97)	1.61 (1.09-2.39)
Head and neck	311	0.58 (0.35-0.97)	0.62 (0.35-1.08)
Lung	238	1.48 (0.85-2.59)	1.46 (0.77-2.77)
Location of first treatment day			
Physician office	514	1.52 (1.12-2.07)	1.36 (0.95-1.95)
Hospital outpatient	298	0.95 (0.63-1.44)	1.08 (0.58-2.02)
Provider NCI designation			
NCI	163	0.56 (0.31-1.01)	0.39 (0.22-0.71)
Non-NCI	649	1.55 (1.18-2.03)	1.52 (1.14-2.04)

Abbreviations: RR, relative risk; NCI, National Cancer Institute designated Comprehensive Cancer Center.

In exploratory subgroup analysis, commercial insurance was associated with greater likelihood of receiving high-price treatment after propensity score weighting among patients with colorectal cancer (risk ratio [RR] 1.61, 95% CI: 1.09-2.39) and those treated by non-NCI providers (RR 1.52, 95% CI 1.14-2.04; Table 4). Commercial insurance was associated with a lower likelihood of receiving high-price treatment among NCI providers (RR 0.39, 95% CI 0.22-0.71).

## Discussion

Among patients diagnosed with colorectal, head and neck, or lung cancer in North Carolina during 2004-2011, those with Medicaid insurance were less likely to receive high-price cancer drugs than their commercially insured counterparts. This difference was reduced after propensity score weighting, suggesting some degree of confounding in the unadjusted estimate. Commercially insured patients remained more likely to receive high-price treatment in the adjusted analysis, although the magnitude of the observed difference was small and within the range where random error may be a possibility.

Likely confounders of the unadjusted association include cancer type and comorbid conditions. Patients with head and neck cancer are less likely to receive high-price treatment^13^ and were over-represented in the Medicaid cohort (Table 2); vice versa for colorectal and lung cancer. Medicaid-insured patients within our cohort were also more likely to have ≥ 2 comorbid conditions which may directly impact drug selection (drug contraindications) as well as ≥ 2 frailty indicators. This is in line with prior observations that comorbid conditions are more common among people insured by Medicaid than those insured by commercial plans.^23^ Oncologists may be less likely to recommend more intensive regimens—such as those with the addition of a biologic agent—to patients they perceive to be frail.

There are several potential contributing explanations for the observed greater use of high-price treatment among commercially insured patients. It may reflect provider responsiveness to the financial incentives presented by drug reimbursement, causing providers to be more likely to use high-price drugs when the billing margin is greater. North Carolina’s Medicaid program reimbursed providers for clinician-administered, “Part B” drugs at the average sales price plus a 6% markup during the study period,^2^ with providers keeping the margin between the reimbursed amount and the drug acquisition cost^4^; commercial insurers typically reimburse at substantially higher rates.^5-7^ Our exploratory finding suggesting that the greater use of high-price treatments for commercially insured patients may be limited to non-NCI (eg, community) providers, consistent with this hypothesis. Community-based oncologists are more likely to have their personal compensation tied to billing, as opposed to the academic setting in which oncologists are typically salaried.^24^

Alternatively, individual providers may manage their Medicaid-insured and commercially insured patients differently for nonfinancial reasons, potentially due to perceptions of Medicaid-insured patients having different values or different abilities to adhere to treatment. Another possibility is patient sorting among providers; Medicaid-insured patients may be more likely to be cared for by providers who are less likely to use high-price treatments in general. Our patient-level analysis is unable to differentiate these 2 potential causes—within-provider differences in treatment by insurance type versus between-provider differences and patient sorting. We partially account for this possibility of between-provider differences by adjusting for NCI versus non-NCI provider status (previously identified as a determinant of high-price treatment),^13^ but a future provider-level analysis in a larger study population would be needed to fully evaluate this hypothesis.

The presence of either within-provider or between-provider differences in cancer care based on insurance status would be a potential cause for concern, warranting further investigation. That Medicaid-insured patients are less likely to receive any care, or appropriate care, for their cancer is a well-known and consistent finding.^25-27^ However, our study suggests potential differences in treatment even for Medicaid-insured patients who have sufficient access to and navigation of the health care system to receive guideline-concordant cancer treatments. The specific high-price treatments included in the current study could be considered “low value for money.” Each of the biologic agents we studied has been found to be beneficial clinically, improving overall survival in some patient subgroups.^28-30^ However, in each case the magnitude of clinical benefit is small relative to the financial cost of these drugs, given their high prices.^31-34^ An awareness of the high prices of these drugs and their commensurate “low value for money” may be a contributing factor as to why the use of these agents by oncologists has remained low; even among commercially insured patients, we observed that oncologists used these drugs only approximately one-third of the time. Therefore, lower receipt of these drugs by Medicaid-insured patients might not raise quality-of-care concerns directly. However, in many cases, more expensive cancer drugs do achieve substantial clinical benefit, such as the newer immunotherapy agents.^35,36^ Whether people insured by Medicaid have lower access to high-price drugs in settings, where those drugs substantially improve long-term outcomes is a critical question for future work.

This study has several important limitations. The study cohort comprised adult, nonelderly patients with cancer who are treated in a single state, and results may not be generalizable to other states, to older patients, or to other insurance types. Relatedly, the number of eligible patients in this single-state population was relatively small, resulting in less-precise estimates. This study included only 3 cancer types, and the results may not apply to the utilization of high-price drugs for other cancer types. The continuous insurance enrollment requirement removed a significant number of patients, and therefore our sample may not fully reflect the population of cancer patients across the socioeconomic spectrum. The observational, nonrandomized design allows for the possibility of confounding by unmeasured variables. Moreover, due to the limited sample size within some of our subgroups of interest, we were unable to balance all measured covariates in all cases, increasing the possibility of confounding bias; these subgroup analyses should be interpreted as exploratory. Finally, more than a decade has elapsed since the time period studied; further research is needed to assess whether these findings would remain true in the current era in which the most commonly used high-price drugs are now immune-oncology agents.

Commercially insured patients with cancer were more likely than those with Medicare to receive high-priced cancer treatments, but this difference appears to be at least partially accounted for by differences in case mix. Further work is needed to assess whether differences in oncology care among payers are driven by patient factors, responsiveness to reimbursement considerations, or a combination of the 2.

## Supplementary Material

oyae035_suppl_Supplementary_Tables_1-4

## Data Availability

The data underlying this article cannot be shared publicly in order to protect the privacy of individuals that participated in the study. The data are housed by the Cancer Information & Population Health Resource (CIPHR) at the University of North Carolina at Chapel Hill and were used for this study under license agreement. The data will be shared on reasonable request and execution of a license agreement with CIPHR.
