# Supplementary material for: Commercial Versus Medicaid Insurance and Use of High-Priced Anticancer Treatments
Source: Oncologist. 2024 Mar 14;29(6):527–33. doi: 10.1093/oncolo/oyae035 (PMC11144993; doi:10.1093/oncolo/oyae035)
Supplement: oyae035_suppl_Supplementary_Tables_1-4 [file oyae035_suppl_supplementary_tables_1-4.docx]

**Commercial vs. Medicaid Insurance and Use of High-Priced Anticancer Treatments: Supplementary Material**

**Supplemental Table 1………………………………………………………………………..pg.2**

**Supplemental Table 2……………………………………………………………………….pg. 3**

**Supplemental Table 3………………………………………………………………………..pg.5**

**Supplemental Table 4………………………………………………………………………..pg.6**

**Supplementary Table 1: Detailed description of selection of cancer types and classification of treatment regimens.** For each cancer type, the study period indicates the range of time for which 1) claims data were available (through 2011), and 2) the high-cost and low-cost treatments under consideration all had FDA approval for that cancer type and stage. Patients were classified according to all drugs received within 60 days following the first drug claim, regardless of the sequencing of those drugs. HC: high-cost; LC: low-cost; 5-FU: 5-fluorouracil.

| Cancer type, stage | Study period | Regimen | Classification |
| --- | --- | --- | --- |
| Colorectal, Stage IV | 2004-11 | [5-FU or capecitabine] + [irinotecan or oxaliplatin] + [bevacizumab, cetuximab, or panitumumab] | HC |
|  |  | [irinotecan or oxaliplatin] + [bevacizumab, cetuximab, or panitumumab] | HC |
|  |  | [5-FU or capecitabine] + [irinotecan or oxaliplatin] | LC |
|  |  | [5-FU or capecitabine] | LC |
|  |  | [irinotecan or oxaliplatin] | LC |
|  |  | bevacizumab, cetuximab, or panitumumab | Excluded |
|  |  | [5-FU or capecitabine] + [bevacizumab, cetuximab, or panitumumab] | Excluded |
| Head & Neck, Stage II-IV | 2006-11 | Cetuximab in combination with any other agent[s] | HC |
|  |  | Any combination of agents, without cetuximab | LC |
| Lung adenocarcinoma, Stage IV | 2006-11 | [carboplatin or cisplatin] + [paclitaxel or pemetrexed] + bevacizumab | HC |
|  |  | [carboplatin or cisplatin] + bevacizumab + [docetaxel, etoposide, gemcitabine, or vinorelbine] | HC |
|  |  | [paclitaxel or pemetrexed] + bevacizumab + [docetaxel, etoposide, gemcitabine, or vinorelbine] | HC |
|  |  | [carboplatin or cisplatin] | LC |
|  |  | [paclitaxel or pemetrexed] | LC |
|  |  | [docetaxel, etoposide, gemcitabine, or vinorelbine] | LC |
|  |  | [paclitaxel or pemetrexed] + [docetaxel, etoposide, gemcitabine, or vinorelbine] | LC |
|  |  | [carboplatin or cisplatin] + [docetaxel, etoposide, gemcitabine, or vinorelbine] | LC |
|  |  | [carboplatin or cisplatin] + [paclitaxel or pemetrexed] | LC |
|  |  | Bevacizumab | Excluded |
|  |  | Bevacizumab + [docetaxel, etoposide, gemcitabine, or vinorelbine] | Excluded |
|  |  | [carboplatin or cisplatin] + bevacizumab | Excluded |
|  |  | [paclitaxel or pemetrexed] + bevacizumab | Excluded |

**Supplementary Table 2: Patient conditions and services included as potential drug contraindications or predictors of frailty.** Conditions are subdivided into “chemotherapy contraindications,” which were derived from the medical conditions identified as “contraindications” or FDA boxed (“black box”) warnings on the manufacturer label for any of the component drugs of the defined low-cost and high-cost treatment regimens, and “frailty indicators,” derived from a previously published algorithm for predicting patient frailty^18^.

| **Condition** | **Associated ICD-9, CPT, or HCPC codes** |
| --- | --- |
| *Chemotherapy contraindications* | |
| Surgery | 00-05.x, 06-07.x, 25-29.x, 30.x, 31.x (EXCEPT 31.4), 32.x, 33.x (EXCEPT 33.2x), 34.x (EXCEPT 34.2x), 35-39.x, 40.x (EXCEPT 40.1x), 41.x (EXCEPT 41.3x), 42-44.x, 45.7x, 45.8x, 46.x, 47.x, 48.x (EXCEPT 48.2x), 49.x (EXCEPT 49.2x), 50.x (EXCEPT 50.1x), 51.x (EXCEPT 51.1x), 52.x (EXCEPT 52.1x), 53.x, 54.1x, 55.x (EXCEPT 55.2x), 56.x (EXCEPT 56.3x), 57.x (EXCEPT 57.3x), 58.x, 59.x, 60.3-60.9, 61-63.x, 64.x (EXCEPT 64.0 and 64.1x), 65.x (EXCEPT 65.1x), 66.x (EXCEPT 66.1x), 67.x (EXCEPT 67.1x), 68.x (EXCEPT 68.1x), 69.x, 70.x (EXCEPT 70.2x), 71.x (EXCEPT 71.1x), 74.x, 76.x (EXCEPT 76.1x), 77.x, 78.x, 79.x, 80-82.x, 83.x (EXCEPT 83.2x), 84.x, 85.x (EXCEPT 85.1x) |
| Hemoptysis | 786.3x |
| Gastrointestinal Bleeding | 456.0, 456.20, 530.7, 530.82, 531.00, 531.01, 531.20, 531.21, 531.40, 531.41, 531.60, 531.61, 532.00, 532.01, 532.20, 532.21, 532.40, 532.41, 532.60, 532.61, 533.00, 533.01, 533.20, 533.21, 533.40, 533.41, 533.60, 533.61, 534.00, 534.01, 534.20, 534.21, 534.40, 534.41, 534.60, 534.61, 569.3, 578.0, 578.1, 578.9 |
| Brain metastasis | 191.x, 198.3x |
| Hearing loss | 388.01, 388.12, 388.2x, 389.x |
| Chronic kidney disease | 5851.x-585.5x, 585.9 |
| End-stage renal disease | 585.6x, 586.x, V451.1 |
| Acute kidney injury | 584.x |
| Cirrhosis | 5714.x-571.9x |
| Viral hepatitis | 070.x |
| *Frailty indicators* | |
| Podiatric care | 700., 703., 681.1 |
| Heart failure | 428., 425., 429.0, 429.1, 429.3, 429.4 |
| Home oxygen | E1390-1392, E0431, E0433-435, E0439, E0441-443 |
| Hypotension or shock | 785.5, 958.4, 998.0, 458. |
| Ambulance transport | A0426, A0427, A0428, A0429, A0999 |
| Stroke/Brain injury | 348., 430., 431., 432., 852., 853., 854., 349.82, 433.01, 433.11, 433.21, 433.31, 433.91, 434.01, 434.11, 434.91 |
| Dementia | 290., 294., 331., 333.90, 333.92, 333.99, 780.93, 438.0, 797 |
| Skin ulcer (decubitis) | 707.0, 707.2, 707. |
| Paralysis | 342., 438.2, 438.3, 438.4, 438.5, 344., 781.4 |
| Wheelchair | E1050, E1060, E1070, E1083-1093, E1100, E1110, E1120, E1140, E1150, E1160, E1161, E1170, K0001-9 |
| Hospital bed | E0250, E0251, E0255, E0256, E0260, E0261, E0265, E0266, E0270, E0290, E0291-297, E0301-304, E0316 |
| Delirium | 29.3, 293.1, 780.97, 780.02, 780.09, 780.39 |
| Falls | E88., E929.3 |
| Hip/Pelvic fractures | 820., 808., V436.4 |
| Physician skilled nursing facility visit | 99304, 99305, 99307, 99308, 99309, 99310, 99315 |
| Walker | E0130, E0135, E0140, E0141, E0143, E0144, E0147, E0148, E0149, E0154, E0155, E0156, E0157, E0158 |
| Malnutrition | 261., 262., 263. |

**Supplementary Table 3: Variables included in determining propensity of exposure weights, overall model and in exploratory subgroup analyses**. NCI, National Cancer Institute.

|  | **Analysis** | | | |
| --- | --- | --- | --- | --- |
| **Variable** | **Overall** | **Cancer type subgroups** | **Location of first treatment subgroups** | **NCI Designation subgroups** |
| Diagnosis year (2-year bins) | X | X | X* | X* |
| Cancer type | X |  | X | X |
| Age (18-44, 45-54, 55-64) | X | X | X |  |
| Surgery | X | X | X |  |
| Gastrointestinal bleeding or hemoptysis | X | X | X |  |
| Brain metastasis | X |  | X |  |
| Race (white vs. non-white) | X |  |  |  |
| Gender (female vs. male) | X |  |  |  |
| Poverty prevalence in patient’s county (<12, 12-14, 14-17, >17) | X |  |  |  |
| Number of frailty indicators (0, 1, >=2) | X |  |  |  |
| Number of drug contraindications (0, 1, >=2) | X |  |  |  |
| Location of first treatment day | X |  |  |  |

*2004-05 and 2006-07 bins combined due to small sample size

**Supplementary Table 4: Characteristics of cohort, by insurance type, in unadjusted data and after inverse probability-of-exposure weighting, in the overall model**. Drug contraindications and frailty indicators are shown in tabulated form because of low frequencies of many individual conditions which may result in possible reidentification due to cell sizes <11; selected contraindications with the greatest theorized potential to affect physician selection of high price vs. low price treatments are shown individually. Standardized mean difference (SMD) reported in terms of the absolute value.

|  | **Unadjusted** | | | | **Weighted** | | |
| --- | --- | --- | --- | --- | --- | --- | --- |
|  |  | **Patient Insurance Type** | |  | **Patient Insurance Type** | |  |
|  | **All patients** | **Medicaid** | **Commercial** | **SMD** | **Medicaid** | **Commercial** | **SMD** |
|  | **(N=812)** | **(n=209, 25.7%)** | **(n=603, 74.3%)** |  | **(n=227.6, 28.0%)** | **(n=585.5, 72.0%)** |  |
| Female gender, n (%) | 310 (38.2) | 81 (38.8) | 229 (38) | 0.02 | 87.7 (38.5) | 220.3 (37.6) | 0.02 |
| Age range, n (%) |  |  |  |  |  |  |  |
| *18-44* | 104 (12.8) | 37 (17.7) | 67 (11.1) | 0.19 | 35 (15.4) | 86.3 (14.7) | 0.02 |
| *45-54* | 304 (37.4) | 73 (34.9) | 231 (38.3) | 0.07 | 91.1 (40.0) | 214.7 (36.7) | 0.07 |
| *55-64* | 404 (49.8) | 99 (47.4) | 305 (50.6) | 0.06 | 101.5 (44.6) | 284.4 (48.6) | 0.08 |
| Non-White race, n (%) | 184 (22.7) | 107 (51.2) | 77 (12.8) | -0.9 | 46.2 (20.3) | 120.1 (20.5) | 0.00 |
| Poverty prevalence in patient’s county (%) |  |  |  |  |  |  |  |
| *<12* | 202 (24.9) | 34 (16.3) | 168 (27.9) | 0.28 | 44.3 (19.5) | 153.7 (26.3) | 0.16 |
| *12-<14* | 185 (22.8) | 39 (18.7) | 146 (24.2) | 0.14 | 82.2 (36.1) | 132.6 (22.7) | 0.30 |
| *14-<17* | 245 (30.2) | 60 (28.7) | 185 (30.7) | 0.04 | 64.2 (28.2) | 184.9 (31.6) | 0.07 |
| *>=17* | 180 (22.2) | 76 (36.4) | 104 (17.2) | 0.44 | 36.8 (16.2) | 114.2 (19.5) | 0.09 |
| Year of Diagnosis, n (%) |  |  |  |  |  |  |  |
| *2004-2005* | 52 (6.4) | 12 (5.7) | 40 (6.6) | 0.04 | 11.3 (5.0) | 35.9 (6.1) | 0.05 |
| *2006-2007* | 189 (23.3) | 43 (20.6) | 146 (24.2) | 0.09 | 49.5 (21.8) | 136.7 (23.3) | 0.04 |
| *2008-2009* | 302 (37.2) | 107 (51.2) | 195 (32.3) | 0.39 | 83.6 (36.8) | 214.4 (36.6) | 0.00 |
| *2010-2011* | 269 (33.1) | 47 (22.5) | 222 (36.8) | 0.32 | 83.1 (36.5) | 198.5 (33.9) | 0.05 |
| Provider NCI designation, n (%) |  |  |  |  |  |  |  |
| *NCI* | 163 (20.1) | 27 (12.9) | 136 (22.6) | 0.25 | 46.4 (20.4) | 120.8 (20.6) | 0.01 |
| *Non-NCI* | 649 (78.4) | 182 (87.1) | 467 (77.4) | 0.25 | 181.2 (79.6) | 464.7 (79.4) | 0.01 |
| Cancer type, n (%) |  |  |  |  |  |  |  |
| *Colorectal* | 263 (32.4) | 54 (25.8) | 209 (34.7) | 0.19 | 79.2 (34.8) | 193.3 (33.0) | 0.04 |
| *Head-and-neck* | 311 (38.3) | 106 (50.7) | 205 (34.0) | 0.34 | 82.2 (36.1) | 219.5 (37.5) | 0.03 |
| *Lung* | 238 (29.3) | 49 (23.4) | 189 (31.3) | 0.18 | 66.1 (29.1) | 172.7 (29.5) | 0.01 |
| Location of first treatment day, n (%) |  |  |  |  |  |  |  |
| *Physician office* | 514 (63.3) | 130 (62.2) | 384 (63.7) | 0.03 | 156.4 (68.7) | 381.2 (65.1) | 0.08 |
| *Hospital outpatient* | 298 (36.7) | 79 (37.8) | 219 (36.3) | 0.03 | 71.2 (31.3) | 204.2 (34.9) | 0.08 |
| Number of drug contraindications, n (%) |  |  |  |  |  |  |  |
| *0* | 290 (35.7) | 73 (34.9) | 217 (36) | 0.02 | 83.4 (36.7) | 207(35.4) | 0.03 |
| *1* | 356 (43.8) | 74 (35.4) | 282 (46.8) | 0.23 | 101.0 (44.4) | 259.3 (44.3) | 0.00 |
| *≥2* | 166 (20.4) | 62 (29.7) | 104 (17.2) | 0.30 | 43.2 (19.0) | 119.2 (20.4) | 0.03 |
| Selected contraindications, n (%) |  |  |  |  |  |  |  |
| *Recent Surgery* | 355 (43.7) | 96 (45.9) | 259 (43.0) | 0.06 | 94.6 (41.6%) | 256.8 (43.9%) | 0.05 |
| *Gastrointestinal bleeding or hemoptysis* | 160 (19.7) | 54 (25.8) | 106 (17.6) | 0.20 | 42.0 (18.5) | 117.9 (20.1) | 0.04 |
| *Brain metastasis* | 76 (9.4) | 16 (7.7) | 60 (10) | 0.08 | 21.0 (9.2) | 54.8 (9.4) | 0.01 |
| Number of frailty indicators, n (%) |  |  |  |  |  |  |  |
| *0* | 541 (66.6%) | 109 (52.2%) | 432 (71.6%) | 0.41 | 165.2 (72.6) | 402.3 (68.7) | 0.09 |
| *1* | 150 (18.5%) | 45 (21.5%) | 105 (17.4%) | 0.10 | 38.1 (16.8) | 114.2 (19.5) | 0.07 |
| *≥2* | 121 (14.9%) | 55 (26.3%) | 66 (10.9%) | 0.40 | 24.2 (10.6) | 69.0 (11.8) | 0.04 |
